# Clinical and Imaging Characteristics in the Diagnosis and Surgical Management of Nipple Discharge Without Clinically Palpable Masses: A Retrospective Cohort Study

**DOI:** 10.1111/1759-7714.70332

**Published:** 2026-06-24

**Authors:** Yang Yang, Ting Lin, Jiaxuan Li, Houpu Yang, Fei Xie, Siyuan Wang, Dingbao Chen, Shu Wang, Lei Chen, Miao Liu

**Affiliations:** ^1^ Breast Disease Center Peking University People's Hospital Beijing China; ^2^ Department of Breast Surgery Tangshan People's Hospital Tangshan China; ^3^ Department of Radiation Oncology Peking University People's Hospital Beijing China; ^4^ Department of Pathology Peking University People's Hospital Beijing China; ^5^ Department of Radiology Peking University People's Hospital Beijing China

**Keywords:** breast cancer, breast MRI, intraductal lesions, nipple discharge

## Abstract

**Background:**

The study aimed to investigate clinical and imaging features in the diagnosis and surgical management of nipple discharge without palpable masses.

**Methods:**

Consecutive patients undergoing surgery for nipple discharge without palpable masses (January 2014–December 2024) were retrospectively enrolled. Clinical, imaging (ultrasound, mammography, MRI), surgical, and pathological data were analyzed. Diagnostic performance of imaging modalities for intraductal lesions/malignancy and their role in incision selection were evaluated. Multivariate regression identified independent risk factors.

**Results:**

Among 664 patients (676 breasts), 512 were pathology‐positive cases. Older age (> 50 years), postmenopausal status, bloody/yellow discharge, and single‐duct involvement correlated with intraductal‐lesions/malignancy (*p* < 0.05). MRI demonstrated superior sensitivity for detecting intraductal‐lesions (81.25%) and cancer (87.32%) versus ultrasound (64.51%/74.26%) and mammography (26.46%/50.39%), with 84.62% concordance in surgical localization. Bloody/yellow discharge, positive ultrasound, and MRI independently predicted intraductal‐lesions (*p* < 0.05).

**Conclusion:**

MRI offers optimal diagnostic sensitivity and precise spatial guidance for surgery. Bloody/yellow discharge, along with positive ultrasound and MRI findings, informs risk stratification and surgical intervention.

## Graphical abstract

Clinical features and multimodal imaging were analyzed for nipple discharge without palpable masses. MRI shows the highest diagnostic sensitivity and reliable surgical localization. Bloody/yellow discharge, along with positive ultrasound and MRI findings, informs risk stratification and surgical intervention.

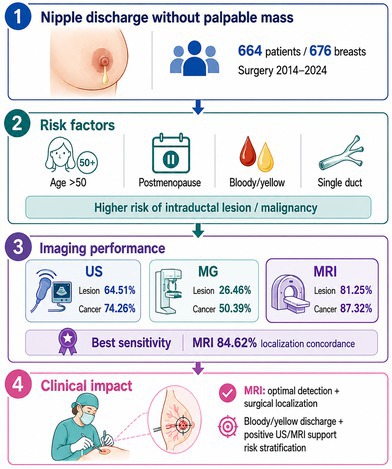

## 1 Introduction

Nipple discharge (ND) is a frequent presenting breast symptom occurring in 2%–5% [1]. Non‐pathological discharge, typically manifesting as bilateral, multi‐ductal serous or milky secretions in reproductive‐aged women, generally requires no intervention [2]. In contrast, pathological nipple discharge refers to spontaneous, unilateral, single‐duct discharge [3], which is often indicative of various intraductal lesions, including both malignant and non‐malignant conditions. Common etiologies include ductal ectasia, intraductal papilloma, atypical hyperplasia, ductal carcinoma in situ, and invasive ductal carcinoma.

The diagnosis of nipple discharge predominantly relied on clinical evaluations and imaging evaluation [2]. Clinical assessments included patient age, the nature of the discharge, and the presence or absence of palpable masses. Imaging evaluations included ultrasound, mammography, galactography, and magnetic resonance imaging (MRI). Previous studies showed that the diagnostic ability of ultrasound for nipple discharge was limited and highly dependent on the technician's skill, with a propensity for missing small lesions [4, 5, 6]. Mammography excelled in calcification detection, while it demonstrated limited sensitivity for small non‐calcified intraductal lesions [7, 8]. Galactography allowed for direct visualization of ductal lesions and helped identify the source of nipple discharge [9, 10]. However, its clinical application is restricted due to challenges in detecting minor ductal branches and the potential for ductal damage during the procedure [11]. In recent years, MRI has been widely used in clinical settings due to its high soft tissue imaging resolution, its ability to image both breasts simultaneously, and its advantage in determining lesion location, morphology, and extent. Previous research showed that MRI possessed high diagnostic sensitivity in cases of nipple discharge ranging from 70% to 100% [12, 13, 14, 15, 16]. Notably, approximately half of the patients with nipple discharge had occult breast lesions that were not detectable through physical examination, ultrasound, and mammography [17]. While MRI could reveal positive findings, including early malignant lesions [14], it is also associated with a high false‐positive rate.

The management of pathological nipple discharge remains contentious and is primarily surgical. Previous studies have revealed that 30%–70% of surgically resected specimens revealed benign pathologies such as mammary duct ectasia and hyperplasia, which do not carry a risk of malignant transformation [18, 19, 20]. This raised concerns about the potential for unnecessary surgical interventions. The incidence of malignancy associated with nipple discharge ranged from 5% to 21% [4], and approximately 5%–17% of intraductal papillomas will eventually progress to malignancy [21]. Lesions associated with nipple discharge did not often present with clinically palpable masses; instead, they tended to be small, multiple, and distributed along affected ducts, resulting in multicentric and multifocal patterns [22]. Preoperative localization presents challenges, and issues such as inadequate exposure of surgical sites, inaccurate resection, and incomplete resection often contribute to recurrence and malignant transformation. MacGrogan et al. [23] reported an overall recurrence rate of 13.4% and a malignization rate of 9.2% among patients with nipple discharge, with a median follow‐up period of 9.2 years.

Accurately assessing the lesion, its location, and the extent of nipple discharge early on is crucial for determining effective treatment and improving prognosis. However, there is still a lack of systematic and comprehensive diagnostic and treatment protocols for patients with nipple discharge without clinically palpable mass. This retrospective cohort study investigated the correlation between clinical and imaging features and pathological outcomes in patients with nipple discharge who do not exhibit clinically palpable masses and analyzed the role of different imaging modalities in guiding the surgical incision selection. It aimed to explore the surgical indications for intraductal lesions and contribute to the development of a rational management framework for this population, enhancing the diagnostic accuracy and optimizing surgical interventions.

## 2 Materials and Methods

### 2.1 Study Design and Patients

This was a retrospective cohort study which was approved by the Ethics Committee of Peking university people's Hospital. Patients who underwent surgical treatment for nipple discharge without clinically palpable masses from January 2014 to December 2024 were consecutively enrolled. Pathological nipple discharge was clinically defined as spontaneous, persistent, bloody, serous, unilateral, or arising from a single duct. The inclusion criteria were as follows: (1) presentation with pathological nipple discharge without palpable breast masses on physical examination; (2) documented preoperative breast imaging evaluation (ultrasound, mammography, or MRI); (3) availability of complete clinical, surgical, and histopathological records. The exclusion criteria were as follows: (1) physiological or drug‐induced discharge (e.g., secondary to pregnancy, lactation, oral contraceptives, or sedatives); (2) history of breast cancer or previous homolateral breast surgical interventions; (3) recent nipple trauma or active acute mastitis at presentation.

### 2.2 Data Collection and Groups

Clinical data were collected for the characteristics and demographics of patients retrospectively, including age, menstrual status, laterality of discharge, color of the discharge (bloody, yellow, serous, or white), number of involved ducts (single duct or multiple ducts), and the duration of the disease course (time from the onset of discharge to the initial medical consultation), and pathological data, including the results of the histopathological examination and immunohistochemical staining.

The pathological results were divided into two groups based on the confirmation of breast cancer or not: the malignancy group and the non‐malignancy group. In terms of the presence or absence of intraductal lesions, patients were divided into two groups: the pathology‐positive group and the pathology‐negative group.

The breast imaging results (ultrasound, mammography and MRI) were categorized according to the Breast Imaging Reporting and Data System (BI‐RADS) established by the American College of Radiology [24]. The imaging diagnoses were dichotomised as follows: BI‐RADS categories 1, 2, and 3 were deemed to be negative to the presence of intraductal lesions related nipple discharge, while BI‐RADS categories 4 and 5 were deemed to be positive to the intraductal lesions related to nipple discharge.

Breast ultrasound examinations were conducted using a GE color Doppler ultrasound diagnostic device, with an L14‐5 transducer and a frequency range of 5–12 MHz. Patients were positioned supine with arms elevated for the examination. The ultrasound examinations were performed by senior physicians, with positive findings defined as BI‐RADS 4 or 5 based on key suggestive features, including hypoechoic lesions in the nipple‐areolar region, ductal dilation with intraductal occupancy, and complex echoic masses on ultrasound images. Conversely, BI‐RADS category 1, 2, or 3 of ultrasound features, such as simple duct ectasia without intraductal occupancy, were classified as negative findings.

Mammographic examinations were performed using a GE Senographe DS digital mammography X‐ray unit, with patients standing for routine bilateral mediolateral oblique (MLO) and craniocaudal (CC) views. The mammograms were interpreted and reviewed by senior radiologists, with positive findings defined as linear or segmental abnormal calcifications along the ducts and occupancy in the nipple‐areolar region on images, indicating the presence of intraductal lesions associated with discharge.

MRI examinations were conducted using a GE 3.0 T Discovery MR 750 W magnetic resonance scanner and a dedicated 8‐channel phased‐array breast coil. Patients were positioned prone with breasts naturally pendant. The scanning sequences included T1‐weighted imaging, T2‐weighted imaging, diffusion‐weighted imaging, and dynamic contrast‐enhanced sequences. The MRI images were interpreted and reviewed by senior radiologists, with positive findings defined as ductal dilation with high signal intensity, high signal intensity in the nipple‐areolar region, and high signal intensity along the ducts on images, suggesting the presence of intraductal lesions related to discharge.

For patients who underwent both breast ultrasound and MRI, two combined diagnostic strategies were further evaluated: the parallel combination model and the serial combination model. In the parallel combination model, a positive result was defined as a positive finding (BI‐RADS 4 or 5) on either ultrasound or MRI; a negative result was defined only when both examinations were negative (BI‐RADS 1–3 on both modalities). In the serial combination model, a positive result was defined only when both ultrasound and MRI were positive (BI‐RADS 4 or 5 on both); otherwise, the result was considered negative.

### 2.3 Lesion Localization and Surgical Information Records

Based on the breast imaging findings, the presence of breast lesions associated with nipple discharge was determined. A horizontal and a vertical line were drawn centered on the nipple, dividing the breast into six regions: outer upper quadrant, outer lower quadrant, inner lower quadrant, inner upper quadrant, directly above, and directly below. Senior radiologists utilized breast imaging to localize the breast lesions into these designated areas. Surgical incision data were obtained from the surgery records, and the position of the surgical incisions was documented as being located in one of six regions.

### 2.4 Statistical Analysis

A statistical analysis was carried out using IBM SPSS Statistics ver. 26.0 (IBM Co., Armonk, NY, USA). Descriptive statistical methods (mean, standard deviation, frequency, percent, minimum and maximum) were used to evaluate the study data. Pearson's chi‐square test was used to compare qualitative data, and Fisher's exact test was applied if the number of subgroups was low. Univariate and multivariate logistic regression analyses were used to analyze the related factors of positive pathology. Sensitivity, specificity, positive predictive value (PPV), and negative predictive value (NPV) were calculated using standard formulas. Based on the results of the analysis, *p* value < 0.05 was considered to be statistically significant.

## 3 Results

### 3.1 Cohort Characteristics

**FIGURE 1:**
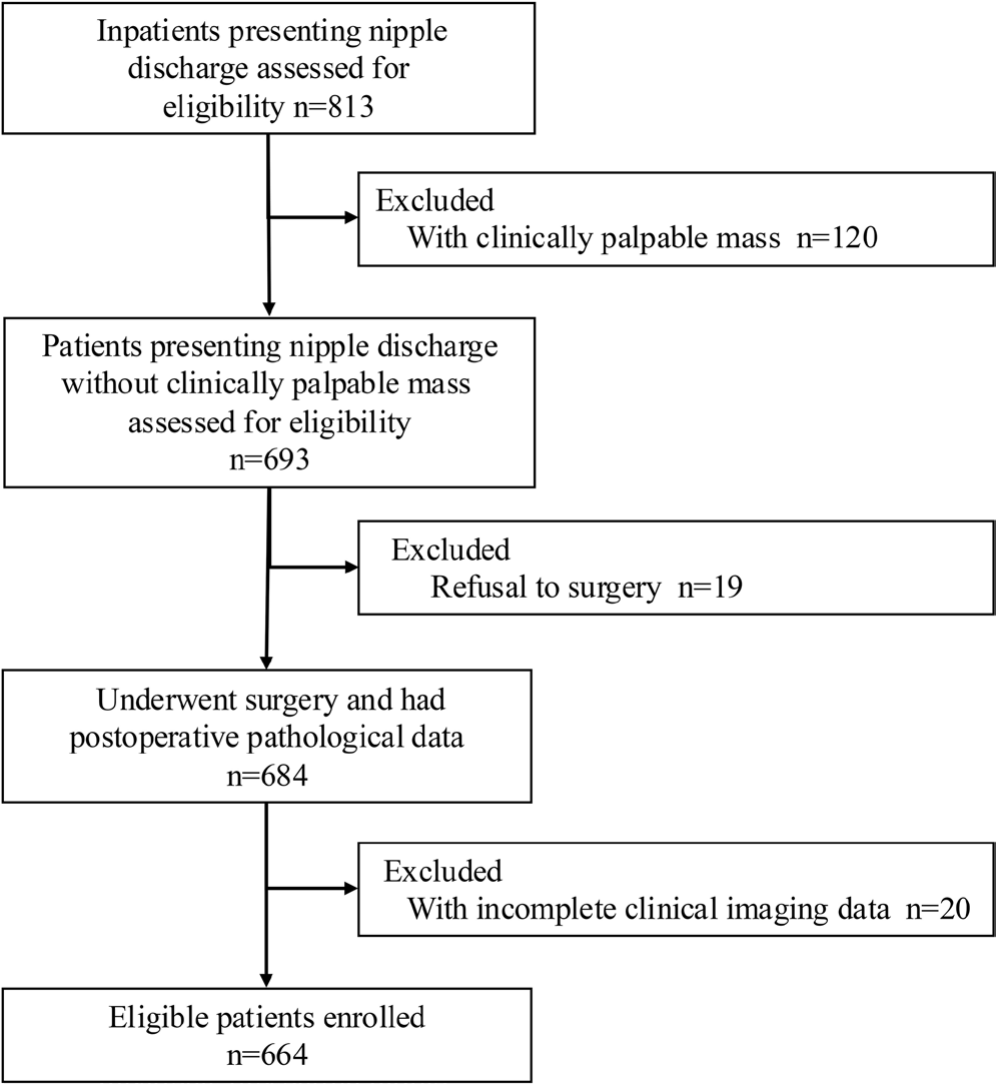
Flow diagram of the study.

A total of 664 patients presenting with nipple discharge without palpable clinical mass who underwent surgical interventions were enrolled in this cohort study, with 12 cases undergoing bilateral breast surgery, thus accounting for 676 affected breasts in total (Figure 1). The median age was 49 years (range, 15–85 years), and the median disease duration was 3 months (range, 1 day to 10 years). 368 patients (55.42%) were premenopausal, and 296 patients (44.58%) were postmenopausal. A total of 652 patients (98.19%) presented with unilateral nipple discharge and 12 patients (1.81%) had bilateral nipple discharge (with the same characteristic of discharge on both sides). 34 patients (5.12%) had multi‐ductal discharge and 630 patients had single duct discharge. Among the 676 affected breasts, there were 320 cases (48.19%) of bloody discharge, 200 cases (30.12%) of yellow discharge, 110 cases (16.57%) of serous discharge, and 34 cases were white discharge (5.12%). Postoperative pathology revealed benign lesions in 530 affected breasts (78.4%) and malignant lesions in 146 affected breasts (21.6%). Based on the postoperative pathology indicating the presence or absence of intraductal lesions, there were 512 in 676 cases (75.74%) in pathology‐positive group and 164 in 676 cases (24.26%) in pathology‐negative group, respectively. The pathology‐positive group included cases of solitary or multiple intraductal papillomas, atypical hyperplasia, intraductal papillomas with atypical hyperplasia, and breast cancer. The pathology‐negative group comprised cases of epithelial hyperplasia, ductal dilation, fibroadenomas, and mastitis‐related inflammatory diseases (Table 1).

**TABLE 1:** Patients characteristics.

Characteristics	*n*/*N* (%)
No. of patients	664 (100)
Age (years)	
≤ 50	357 (53.77)
> 50	307 (46.23)
Menstrual status	
Premenopause	368 (55.42)
Postmenopause	296 (44.58)
Laterality	
Unilateral	652 (98.19)
Bilateral	12 (1.81)
Ductal involvement	
Single duct	630 (94.88)
Multiple ducts	34 (5.12)
Color	
Bloody	320 (48.19)
Yellow	200 (30.12)
Serous	110 (16.57)
White	34 (5.12)
Duration of the disease course (month)	
≤ 1	202 (30.42)
1–3	134 (20.18)
3–13	225 (33.89)
> 12	103 (15.51)
Pathology (664patients, 676 cases)	
Epithelial hyperplasia	70 (10.36)
Ductal dilation	74 (10.95)
Fibroadenoma	11 (1.63)
Mastitis and inflammatory diseases	9 (1.33)
Solitary intraductal papilloma	128 (18.93)
Multiple intraductal papillomas	27 (3.99)
Atypical hyperplasia	24 (3.55)
Intraductal papilloma with atypical hyperplasia	187 (27.66)
Breast cancer	146 (21.60)
Ductal carcinoma in situ (DCIS)	47 (6.95)
Solid papillary carcinoma	36 (5.33)
Intraductal papillary carcinoma	16 (2.37)
Invasive breast carcinoma	47 (6.95)

### 3.2 Relationship Between Clinical Characteristics and Pathological Features

Among the clinical factors, age (*p* < 0.001), menstrual status (*p* < 0.001), and discharge color (*p* < 0.001) were significantly different between the non‐malignant and malignant groups. There was no significant difference between bloody discharge and yellow discharge in identifying breast cancer (*p* > 0.05), while other colored discharges exhibited significant differences when compared with both bloody and yellow discharges (*p* < 0.05) (Table S1). Of 528 affected breasts with bloody or yellow discharge, there were 137 malignancies. Of 148 affected breasts with other colored discharges, there were 9 malignancies. Out of 322 affected breasts with bloody discharge, 89 were pathologically confirmed as malignant. The diagnostic performance yielded a sensitivity of 60.96% (89/146), specificity of 56.03% (297/530), PPV of 27.64% (89/322), and NPV of 83.90% (297/354). Among 206 breasts with yellow discharge, 48 had malignant lesions. Yellow discharge demonstrated a sensitivity of 32.88% (48/146), specificity of 70.19% (372/530), PPV of 23.30% (48/206), and NPV of 79.15% (372/470).

Similarly, age (*p* < 0.001), menstrual status (*p* < 0.001), number of involved ducts (*p* < 0.001), and discharge color (*p* < 0.001) were significantly different between the pathologically positive group and the pathologically negative group (Table S2). Bloody and yellow discharges accounted for 88.67% (454/512) of the pathologically positive group. Out of 322 patients with bloody discharge, a majority of 91.30% (294/322) had pathologically positive lesions. The diagnostic performance of bloody discharge for identifying pathologically positive lesions was as follows: sensitivity 57.42% (294/512), specificity 82.93% (136/164), PPV 91.30% (294/322), and the NPV 38.42% (136/354). Among 206 breasts exhibiting yellow discharge, 160 were confirmed as pathologically positive. The diagnostic performance for identifying pathologically positive lesions was as follows: sensitivity 31.25% (160/512), specificity 71.95% (118/164), PPV 77.67% (160/206), and the NPV 25.11% (118/470).

### 3.3 Breast Imaging Diagnostic Performance Analysis

Of the 676 affected breasts, there were 42, 91, and 354 cases that did not undergo ultrasound, mammography, and MRI. A total of 634, 585, and 322 cases were included to analyze the diagnostic efficiency of breast imaging.

Ultrasound profiles showed that there were 55.84% (354/634) of cases with positive findings and 44.16% (280/634) of cases with negative findings. Among the 479 pathologically positive lesions, 309 cases were reported as positive by ultrasound. Among the 155 pathologically negative lesions, 45 cases were reported as positive. Positive ultrasound significantly differed between the pathology‐positive and pathology‐negative groups (*p* < 0.001) (Table S3). The sensitivity, specificity, PPV, and NPV of ultrasound diagnosis for intraductal lesions were 64.51%, 70.97%, 87.29%, and 39.29%, respectively. Of the 136 cases with breast cancer, 74.26% (101/136) were reported as BI‐RADS category ≥ 4. In the non‐malignancy group, 34.74% (173/498) of cases were reported as BI‐RADS category ≥ 4. There was a significant difference in BI‐RADS categories between the two groups (*p* < 0.001) (Table S4). The sensitivity, specificity, PPV, and NPV of ultrasound diagnosis for breast cancer were 74.26%, 65.26%, 36.86%, and 90.28%, respectively.

Mammography profiles showed 24.96% (146/585) of cases had positive report and 75.04% (439/585) cases had negative reports. Positive mammography did not significantly differ between the pathology‐positive and pathology‐negative groups (*p* = 0.133) (Table S3). The sensitivity, specificity, PPV, and NPV of mammography diagnosis for intraductal lesions were 24.46%, 79.86%, 80.82%, and 25.28%, respectively. Of the 129 cases with breast cancer, 50.39% (65/129) were reported as BI‐RADS category ≥ 4. In the non‐malignancy group, 17.98% (82/456) of cases were reported as BI‐RADS category ≥ 4. There was a significant difference in BI‐RADS categories between the two groups for positive mammography (*p* < 0.001) (Table S4). The sensitivity, specificity, PPV, and NPV of mammography diagnosis for breast cancer were 50.39%, 82.02%, 44.22%, and 85.39%, respectively.

MRI profiles showed 72.98% (235/322) of cases had positive report and 27.02% (87/322) cases had negative report. Positive MRI significantly differed between the pathology‐positive and pathology‐negative groups (*p* < 0.001) (Table S3). The sensitivity, specificity, PPV, and NPV of MRI diagnosis for intraductal lesions were 81.25%, 51.22%, 82.98%, and 48.28% respectively. Among the 80 cases with negative findings on both ultrasound and mammography, the sensitivity, specificity, PPV, and NPV of MRI diagnosis for intraductal lesions were 80.00%, 33.33%, 66.67%, and 50.00% respectively. Of the 71 cases with breast cancer, 87.32% (62/71) were reported as BI‐RADS category ≥ 4. In the non‐malignancy group, 55.78% (140/251) of cases were reported as BI‐RADS category ≥ 4. There was a significant difference in BI‐RADS category between the two groups for positive MRI (*p* < 0.001) (Table S4). The sensitivity, specificity, PPV, and NPV of MRI diagnosis for breast cancer were 87.32%, 44.22%, 30.69%, and 92.50%, respectively.

To explore the multi‐modal synergistic diagnostic value, a subgroup analysis was performed in 307 patients who underwent both breast ultrasound and MRI. Parallel (either‐positive) and serial (both‐positive) combined models were further evaluated. For pathological positivity, the parallel model achieved a sensitivity of 92.11%, specificity of 37.97%, PPV of 81.08%, and NPV of 62.50%. The serial model showed lower sensitivity of 59.21% but markedly elevated specificity of 89.87% and PPV of 94.41%, with an NPV of 43.29% (Table 2A). For malignant lesions, the parallel model presented 100% sensitivity and 100% NPV, along with a specificity of 20.17% and PPV of 26.64%. The serial model maintained balanced diagnostic performance, with sensitivity of 79.71%, specificity of 63.03%, PPV of 38.46%, and NPV of 91.46% (Table 2B).

**TABLE 2A:** Analysis of the correlation between positive imaging and pathological positivity.

Imaging	Pathologically positivity			
	Sensitivity (%)	Specificity (%)	PPV (%)	NPV (%)
Ultrasound	64.51	70.97	87.29	39.29
(*n* = 634)	(309/479)	(110/155)	(309/354)	(110/280)
Mammography	26.46	79.86	80.82	25.28
(*n* = 585)	(118/446)	(111/139)	(118/146)	(111/439)
MRI	81.25	51.22	82.98	48.28
(*n* = 322)	(195/240)	(42/82)	(195/235)	(42/87)
Ultrasound/MRI (Parallel model)	92.11	37.97	81.08	62.50
(*n* = 307)	(210/259)	(30/79)	(210/259)	(30/48)
Ultrasound/MRI	59.21	89.87	94.41	43.29
Serial model (*n* = 307)	(135/228)	(71/79)	(135/143)	(71/164)

*Note:* MRI: magnetic resonance imaging. Parallel model: a combined positive result was defined as a BI‐RADS ≥ 4 finding on either ultrasound or MRI. Serial model: a combined positive result was defined as a BI‐RADS ≥ 4 finding on both ultrasound and MRI. PPV: positive predictive value. NPV: negative predictive value.

**TABLE 2B:** Analysis of the correlation between positive imaging and malignancy.

Imaging	Malignancy			
	Sensitivity (%)	Specificity (%)	PPV (%)	NPV (%)
Ultrasound	72.46	65.26	36.86	90.28
(*n* = 634)	(101/136)	(325/498)	(101/274)	(325/360)
Mammography	50.39	82.02	44.22	85.39
(*n* = 585)	(65/129)	(374/456)	(65/147)	(374/438)
MRI	87.32	44.22	30.69	92.50
(*n* = 322)	(62/71)	(111/251)	(62/202)	(111/120)
Ultrasound/MRI	100.00	20.17	26.64	100.00
Parallel model (*n* = 307)	(69/69)	(48/238)	(69/259)	(48/48)
Ultrasound/MRI	79.71	63.03	38.46	91.46
Serial model (*n* = 307)	(55/69)	(150/238)	(55/143)	(150/164)

*Note:* MRI: magnetic resonance imaging. NPV: negative predictive value. Parallel model: a combined positive result was defined as a BI‐RADS ≥ 4 finding on either ultrasound or MRI. PPV: positive predictive value. Serial model: a combined positive result was defined as a BI‐RADS ≥ 4 finding on both ultrasound and MRI.

### 3.4 Comparison of the Consistency Between Breast Lesion Locations Indicated by Different Imaging Evaluations and the Selection of Surgical Procedure

Of the 676 affected breasts, the location of lesion and surgical incision were retrospectively collected. 51.89% (329/634) of lesion locations were clearly identified by ultrasound and 22.74% (133/385) of lesion locations were identified by mammography. In contrast, MRI provided clear lesion locations in 70.50% (227/322) of the cases, which was higher than that of ultrasound and mammography. A total of 219 patients with complete clinical records, breast imaging profile (ultrasound, mammography, and MRI) and pathological data were collected to compare the consistency between lesion locations indicated by different imaging examinations and the selection of surgical incision location. Surgical incision distributions were as follows: 6.94% (59/219) in the outer upper quadrant, 16.44% (36/219) in the outer lower quadrant, 12.33% (27/219) in the inner upper, 12.33% (27/219) in the inner lower quadrants, 19.18% (42/219) in directly above and 12.79% (28/219) in directly below. The consistency rate of lesion location and incision location indicated by MRI was 84.62% (44/52), higher than 70.80% (80/113) by ultrasound and 59.18% (29/49) by mammography.

**FIGURE 2:**
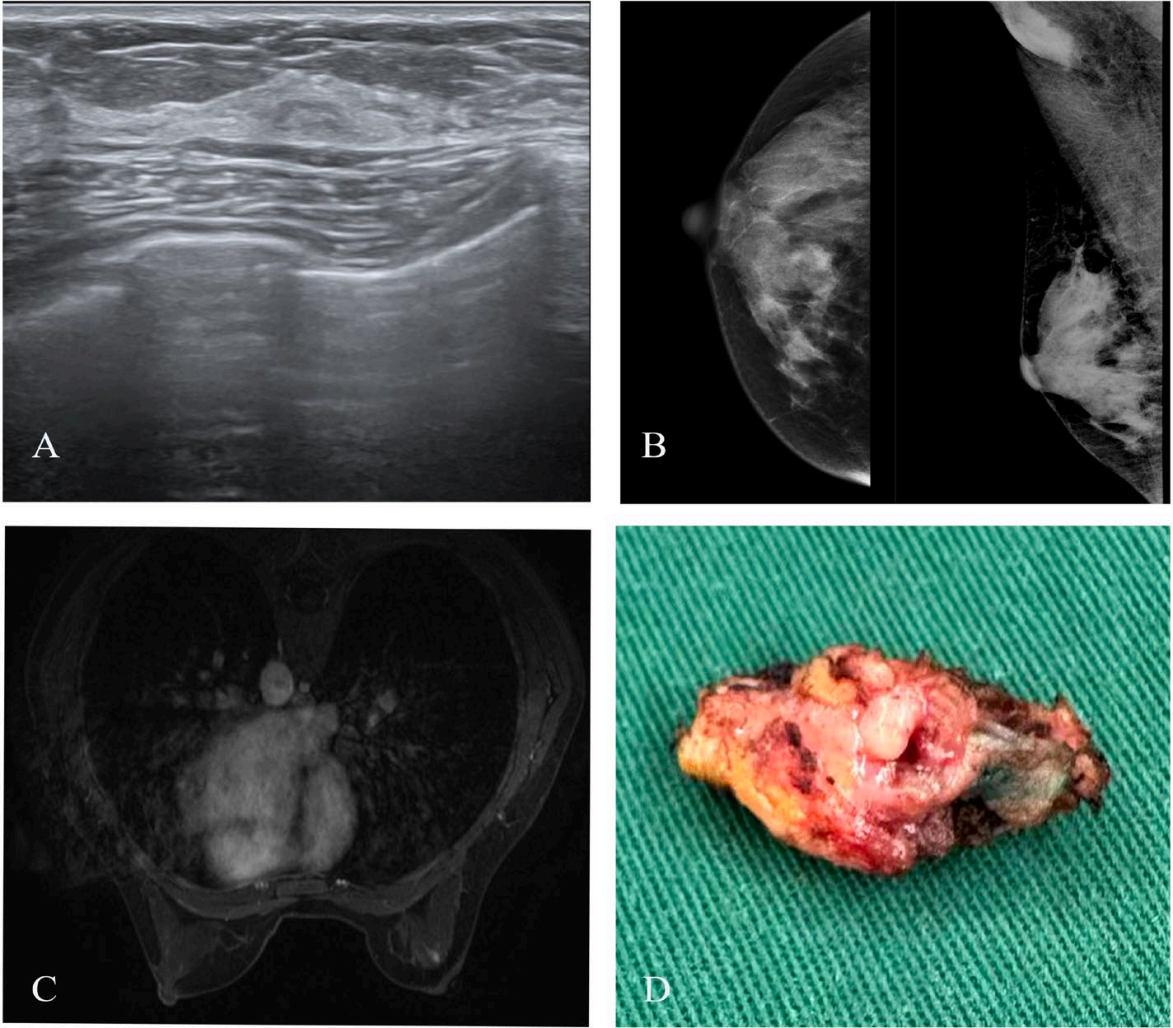
Imaging and postoperative specimen of patient 1 (A) no abnormality detected on breast ultrasound; (B) no abnormality detected on mammography in craniocaudal (CC) and mediolateral oblique (MLO) views; (C) focal enhancing lesion posterior to the nipple in the right breast on contrast‐enhanced scan; (D) surgically resected specimen.

**FIGURE 3:**
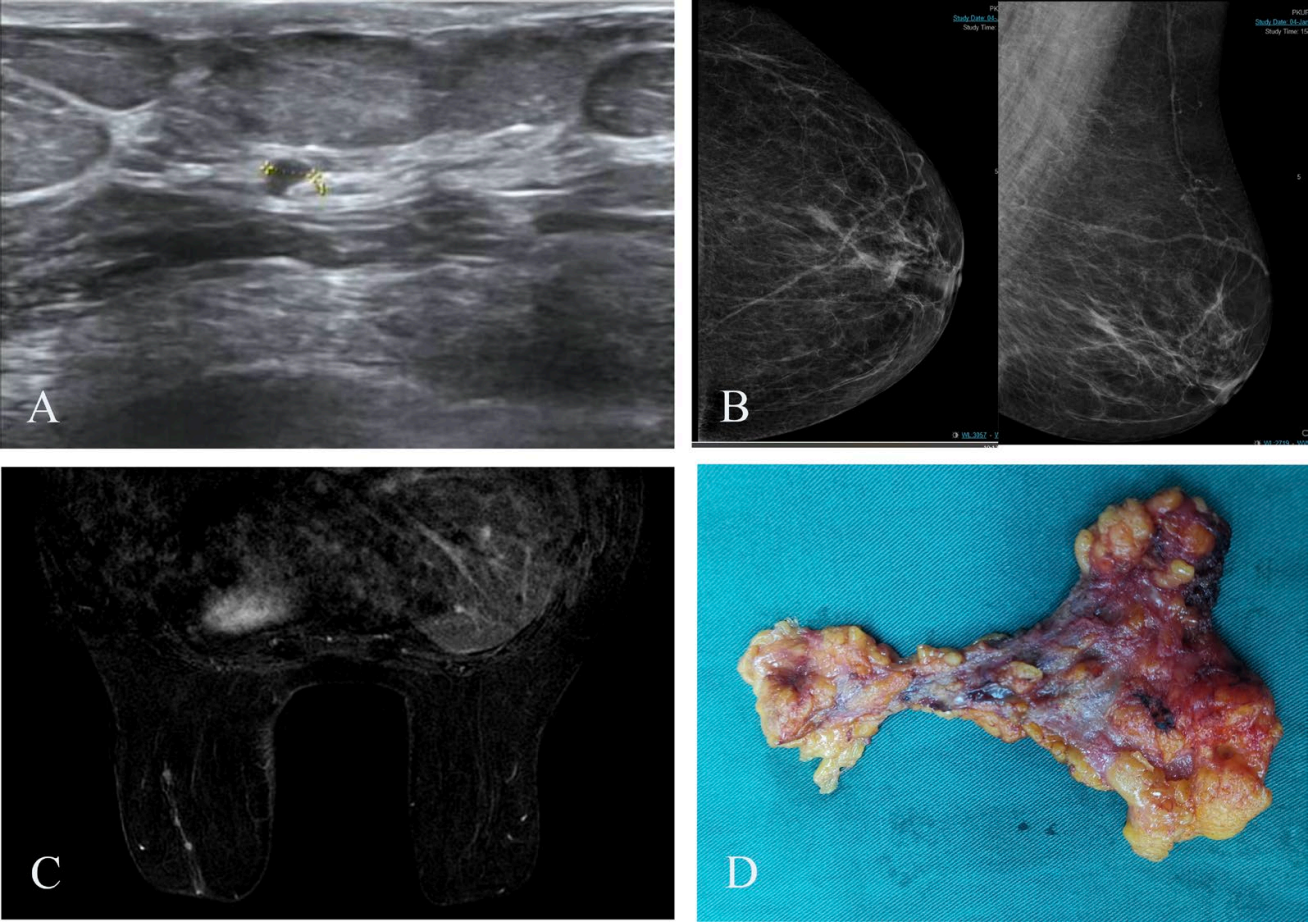
Imaging and postoperative specimen of patient 2 (A) Hypoechoic nodule with irregular shape on breast ultrasound; (B) No abnormalities detected on mammography in craniocaudal (CC) and mediolateral oblique (MLO) views; (C) left breast nodule with ductal dilation on contrast‐enhanced scan; (D) surgically resected specimen.

MRI not only helped to determine the location of lesions, but also guided the selection of surgical incisions and the extent of surgical resection. For example, patient 1, a 35‐year‐old female, presented with a two‐month history of unilateral bloody discharge from the right breast; both ultrasound and mammography showed no abnormalities. MRI revealed a small focal enhancement area behind the right nipple, measuring 0.7 × 0.6 cm, with a plateau‐type enhancement curve. An areolar arc incision beneath the nipple was made and the lesion was accurately located. The extent of resection was well guided, with the specimen measuring about 3 × 2 cm. A central grayish‐white lesion of 0.4 cm in diameter was pathologically confirmed as solitary intraductal papilloma (Figure 2). Patient 2, a 67‐year‐old female, presented with a one‐month history of unilateral bloody nipple discharge from the left breast. Mammography was negative, and ultrasound showed a hypoechoic area of 0.35 × 0.3 cm in the lower outer quadrant. MRI demonstrated dilated ducts in the lateral aspect and enhancing lesions distributed along the ducts, suggesting extensive involvement of the lesion. A lateral radiating incision was made for surgery, and the complete lesion was accurately excised. The resected specimen measured about 5 × 4 cm, with a grayish‐white and grayish‐yellow appearance, and no nodules or masses were observed. Further extensive sampling was performed and postoperative pathology indicated intraductal papilloma with atypical hyperplasia (Figure 3).

### 3.5 Risk Factors Analysis on Surgical Indications

A total of 219 patients with complete clinical records, breast imaging profile (ultrasound, mammography, and MRI), and pathological data were analyzed to investigate the factors associated with surgical indications for nipple discharge disease without clinically palpable mass. Univariate regression analysis showed that bloody or yellow discharge, positive ultrasound, and positive MRI were significantly associated with pathological positivity, indicating the intraduct lesions (*p* < 0.05). Age, menstrual status, number of involved ducts, disease duration, and mammography had no significant association with pathologically positive lesions (*p* > 0.05) (Table 3). Multivariate analysis by logistic regression showed that blood discharge (OR = 22.86, *p* < 0.001), yellow discharge (OR = 4.39, *p* = 0.002), positive ultrasound (OR = 0.15, *p* < 0.001), and positive MRI (OR = 0.28, *p* = 0.008) were independent risk factors for the intraduct lesions (Table 4).

**TABLE 3:** Univariate analysis of the correlation between clinical characteristics and pathological positivity in patients with nipple discharge without clinically palpable mass (*n* = 219).

Characteristics	Pathological positivity *n* (%)	Pathological negativity *n* (%)	Univariate regression analysis	
	*N* = 172	*N* = 47	OR (95% CI)	*p* * value
Age (years)			1.47 (0.76–2.84)	0.254
≤ 50	90 (52.33)	29 (61.70)		
> 50	82 (47.67)	18 (38.30)		
Menstrual status			0.61 (0.31–1.19)	0.147
Pre‐menopause	93 (54.07)	16 (34.04)		
Post‐menopause	79 (45.93)	31 (65.96)		
Ductal involvement			0.32 (0.08–1.25)	0.102
Single duct	167 (97.09)	43 (91.49)		
Multiple	5 (2.91)	4 (8.51)		
Color				
Bloody	98 (56.98)	6 (12.77)	23.59 (8.51–65.43)	< 0.001
Yellow	56 (32.56)	15 (31.91)	5.39 (2.36–12.34)	< 0.001
Others^a^	18 (10.46)	26 (55.32)	1.00	
Duration of disease (month)				
≤ 1	51 (29.65)	12 (25.53)	1.17 (0.43–3.20)	0.756
1–3	30 (17.44)	5 (10.64)	1.66 (0.49–5.65)	0.421
3–12	62 (36.05)	22 (46.81)	0.78 (0.31–1.95)	0.592
> 12	29 (16.86)	8 (17.02)	1.00	
Ultrasound			0.14 (0.07–0.30)	< 0.001
Positive	118 (68.60)	11 (23.40)		
Negative	54 (31.40)	36 (76.60)		
Mammography			0.81 (0.36–1.82)	0.605
Positive	39 (22.67)	9 (19.15)		
Negative	133 (77.33)	38 (80.85)		
MRI			0.33 (0.16–0.68)	0.003
Positive	145 (84.30)	30 (63.83)		
Negative	27 (15.70)	17 (36.17)		

^a^ Others: Serous or white nipple discharge. CI: confidence interval. MRI: magnetic resonance imaging. OR: Odds Ratio.

* *p* values and 95% CIs were determined using univariate logistic regression analysis via the Wald test. *p* < 0.05 was considered statistically significant.

**TABLE 4:** Multivariate logistic regression analysis to determine the risk factors associated with pathologic nipple discharge in pathological positivity (*n* = 219).

Characteristics	Pathological positivity *n* (%)	Pathological negativity *n* (%)	Multivariate logistic regression analysis	
	*N* = 172	*N* = 47	OR (95% CI)	*p* * value
Color				
Bloody	98 (56.98)	6 (12.77)	22.86 (7.43–70.33)	< 0.001
Yellow	56 (32.56)	15 (31.91)	4.39 (1.72–11.21)	0.002
Others^a^	18 (10.46)	26 (55.32)	1.00	
Ultrasound			0.15 (0.07–0.36)	< 0.001
Positive	118 (68.60)	11 (23.40)		
Negative	54 (31.40)	36 (76.60)		
MRI			0.28 (0.11–0.72)	0.008
Positive	145 (84.30)	30 (63.83)		
Negative	27 (15.70)	17 (36.17)		

^a^ Others: Serous or white nipple discharge. CI: confidence interval. MRI: magnetic resonance imaging. OR: odds ratio.

* *p* values and 95% CIs were determined using multivariate logistic regression analysis via the Wald test. *p* < 0.05 was considered statistically significant.

## 4 Discussion

Nipple discharge is the third most common breast complaint after pain and a lump. The most common causes of pathologic nipple discharge are benign, such as intraductal papilloma/papillomatosis (35%–48%) and duct ectasia (17%–36%) [25, 26]. However, 5%–12% of patients with breast cancer presented with pathological nipple discharge as the sole symptom [27]. The diagnosis for patients with nipple discharge and no palpable masses commonly included the color of the discharge, ultrasound, and mammography, which had low positive rates and sensitivity and may lead to delayed treatment or overtreatment. The challenge for clinicians is to explore diagnostic and surgical procedures available to identify intraductal lesions or breast cancer in patients presenting with nipple discharge without a clinically palpable mass. In this study, 664 patients with 676 affected breasts who had nipple discharge without clinically palpable masses were retrospectively included in clinicopathological correlation analysis. It aims to explore the clinical features and surgical indications in this population, with the expectation of establishing a rational diagnostic and treatment process and optimizing therapeutic scheme selection.

Previous studies indicated that old age (> 50 years) was a high‐risk factor for breast malignancy. It should be especially heightened vigilance for early intraductal malignant changes in postmenopausal patients > 50 years old with nipple discharge [27, 28]. In this study, there were 146 malignancies (21.6%) and 530 non‐malignancies (78.40%). Among them, old age accounted for 67.81% in the malignancy group and 40.19% in the non‐malignancy group. Postmenopausal patients accounted for 65.07% in the malignancy group and 38.49% in the non‐malignancy group. Consistent with previous studies, the results indicated that age over 50 years old and post‐menopause were high‐risk factors for malignant lesions (*p* < 0.001). A meta‐analysis study showed that patients with bloody discharge had a higher risk of breast cancer than those with other types of discharge [29]. Especially, unilateral single‐duct bloody discharge often indicated breast cancer [30]. In this study, the proportion of bloody and yellow discharge in the malignancy group was 60.96% and 32.88%. While in the benign lesion group, it was 43.96% and 29.81%, respectively, suggesting that bloody or yellow discharge were risk factors of malignancy (*p* < 0.001). The current study revealed that bloody discharge served as the primary sensitivity alarm for underlying malignancy, and bloody or yellow discharge delivered a superior specificity that justifies safe clinical surveillance. However, there were also reports in the literature that the nature of the discharge was not associated with breast cancer [31]. The clinical value of bloody discharge in distinguishing between benign and malignant lesions was limited [32, 33]. The sensitivity of bloody discharge for the diagnosis of breast cancer was high, while the specificity was low. The surgical indication may be overtreated based on the color of discharge alone.

The breast imaging evaluation for nipple discharge includes ultrasound, mammography and MRI [12, 34]. In this study, a total of 354 cases showed positive findings on ultrasound, with 309 (87.3%) pathologically positive lesions, indicating that positive ultrasound findings were a higher‐risk factor of intraductal papillomas, atypical hyperplasia, and breast cancer (*p* = 0.000). The sensitivity, specificity, PPV and NPV of ultrasound for diagnosing pathologically positive lesions was 64.51%, 70.97%, 87.29% and 39.29%, which was consistent with previous studies [7]. Among the 274 affected breasts with BI‐RADS ≥ 4, 101 cases (74.26%) were identified in the malignancy group and 173 cases (34.74%) were benign lesions, indicating that patients with nipple discharge and BI‐RADS ≥ 4 are at a higher risk of malignancy (*p* < 0.001), which was consistent with previous studies [2, 7, 8]. The sensitivity and specificity of BI‐RADS ≥ 4 for diagnosing breast cancer was 74.26% and 65.26%. Combining previous studies and this study, ultrasound has a predictive diagnostic value for intraduct lesions with nipple discharge without clinically palpable mass.

Mammography plays a significant role in the early detection of breast cancer. However, its diagnostic value for nipple discharge was limited, with a sensitivity of only 7%–26% [8, 12, 34]. In this study, 75.04% of the affected breasts showed no significant abnormalities on mammography, and the positive rate of mammography was limited for the diagnosis of intraductal lesions. This may be attributed to the small size of the lesions and their frequent occurrence in the nipple‐areolar region, where glandular tissue is dense. In this study, there were a total of 129 affected breasts with breast cancer, and 50.39% (65/129) of these had a BI‐RADS ≥ 4, while 17.89% of the benign lesion breasts had a BI‐RADS ≥ 4 (*p* < 0.001), indicating that a mammographic BI‐RADS ≥ 4 was a high‐risk factor for breast cancer. The sensitivity was 50.39%, and the specificity was 82.02%, which was generally in line with previous reports [2, 7, 35].

MRI has high soft tissue resolution, which confers an advantage in the detection and diagnosis of lesions in patients with nipple discharge. Particularly, for patients with nipple discharge and no clinically palpable masses, MRI is more capable of revealing non‐mass enhancements, substantially improving the detection rate of early and multicentric lesions, and has gradually been applied in the diagnostic evaluation of nipple discharge in recent years [36]. Previous studies showed that the sensitivity and specificity of MRI for detecting intraductal lesions were 70%–100% and 37%–97% [12, 13, 14, 15, 16, 34]. In this study, the sensitivity of positive MRI findings for the detection of intraductal lesions was 81.25%, with a specificity of 51.22%, a PPV of 82.98%, and a NPV of 48.48%. 202 affected breasts had MRI with a BI‐RADS ≥ 4, of which 62 were breast cancers, accounting for 87.32% (62/71) of the malignancy group, and 140 were benign lesions, accounting for 55.78% (140/251) of the non‐malignancy group. This suggested that patients with nipple discharge and BI‐RADS ≥ 4 had a higher risk of breast cancer (*p* < 0.001). The sensitivity of MRI with BI‐RADS ≥ 4 for diagnosing malignancy in affected breasts with nipple discharge was 87.32%, with a specificity of 44.22%, a PPV of 30.69%, and a NPV of 92.50%. This indicated that MRI had high diagnostic sensitivity, which was generally in line with previous reports that the sensitivity and specificity of MRI in detecting breast cancer were 100% (11/11) and 68% (54/80), respectively [15]. However, in another study, the sensitivity of MRI for breast cancer was only 40%, suggesting its limited value [37]. The difference in study populations may account for the variation in these results.

In this study, the sensitivity of MRI for both malignant lesions and positive pathology in patients with nipple discharge was better than that of ultrasound and mammography. Previous studies showed that MRI could effectively display ductal lesions, with high sensitivity and NPV, making it an important option for patients with nipple discharge who have negative findings of conventional imaging [38]. Zacharioudakis et al. analyzed 82 patients with negative ultrasound and mammography but with epithelial cells or blood in nipple discharge cytology smears, finding that the sensitivity and specificity of MRI for diagnosing occult malignant lesions were 85.71% and 98.53%, with PPV and NPV of 92.31% and 97.1%, respectively [14]. In this study, for patients with occult ultrasound and mammography, the diagnostic sensitivity of breast MRI for pathological positivity was 80%, with a specificity of 33.33%, a PPV of 66.67%, and a NPV of 50%, suggesting that when the conventional imaging was negative, MRI was optimal for diagnosing intraductal lesions.

MRI alone exhibited relatively high sensitivity but low specificity, which inevitably led to an elevated false‐positive rate. The main underlying reason is that many benign breast lesions may present similar morphological and signal characteristics on MRI, causing unavoidable imaging overlap with malignant lesions and resulting in false‐positive misjudgment. Given that both ultrasound and MRI possess independent diagnostic advantages, we further performed a subgroup analysis of patients who completed both examinations. Compared with single‐modality imaging, the parallel (either‐positive) combination exhibited an exceptional rule‐out capacity, maximizing the screening sensitivity and NPV to 92.11% and 62.50% for pathological positivity, respectively, while achieving an optimal 100% for both metrics in malignancy identification. Conversely, the serial (both‐positive) combination effectively mitigated the high false‐positive rates associated with standalone MRI, elevating the diagnostic specificity from 51.22% to 89.87% for pathological positivity and from 44.22% to 63.03% for malignancy, respectively. These support that the parallel approach is preferable for screening and malignant lesion exclusion, whereas the serial strategy provides higher diagnostic certainty to guide precise clinical management and surgical decision‐making, thereby minimizing the interference of benign lesions and lowering unnecessary positive judgments.

Surgery was the primary treatment for nipple discharge, including the resection of the affected glandular tissue and masses centered around the affected ducts, and the surgical extent required precise localization. However, diseases associated with nipple discharge were complex, frequently lacking clinically palpable masses and even negative imaging results, leading to difficulties in preoperative localization and incomplete surgical resection. Currently, clinical practices often employed dyes such as methylene blue and nanocarbon as markers to aid in localizing the lesion, but the method was limitedly applicable to patients with preoperative nipple discharge and was associated with issues such as the blue dyed surgical field of view and the expanding surgical resection tissues. This study compared the assessment of lesion location in nipple discharge by ultrasound, mammography, and MRI on the selection of surgical incision, aiming to explore the value of different imaging evaluations in preoperative localization. Previous research showed that MRI, with its high soft tissue resolution, thin scanning sections, and multiparameter multiplanar imaging capabilities, was beneficial for determining the location and extent of lesions. MRI could clearly display both mass‐like lesions (single and multiple) and non‐mass‐like lesions (ducts and areas), allowing for more accurate localization of lesions, determination of resection extent, and guidance of surgery [39]. Its value in accurately assessing the extent of lesions had also been indicated in peritoneal cancer [40]. In this study, the consistency between MRI assisted determination of lesion location in the discharging and the selection of surgical incision was significantly higher than that of ultrasound and mammography, suggesting that MRI may be more advantageous for the selection of surgical incisions. However, this study was a retrospective analysis with limited data completeness and differences in the enrolled population, and further confirmation with large‐sample studies is needed in future.

In 2017, the American College of Radiology (ACR) published appropriateness criteria for the evaluation of nipple discharge, which were further updated and refined in 2022 [4, 12, 24, 34]. However, the existing criteria do not differentiate between patients with nipple discharge who do or do not have clinically palpable masses. For the patients with nipple discharge without a palpable mass, appropriate diagnostic and therapeutic processes require further investigation. This study revealed that bloody discharge, yellow discharge, positive breast ultrasound, and positive MRI were all independent risk factors for suffering from breast diseases such as intraductal papillomas, atypical hyperplasia, and breast cancer. Mammography, age, menstrual status, number of discharge ducts, and disease duration had no significant correlation with breast disease.

**FIGURE 4:**
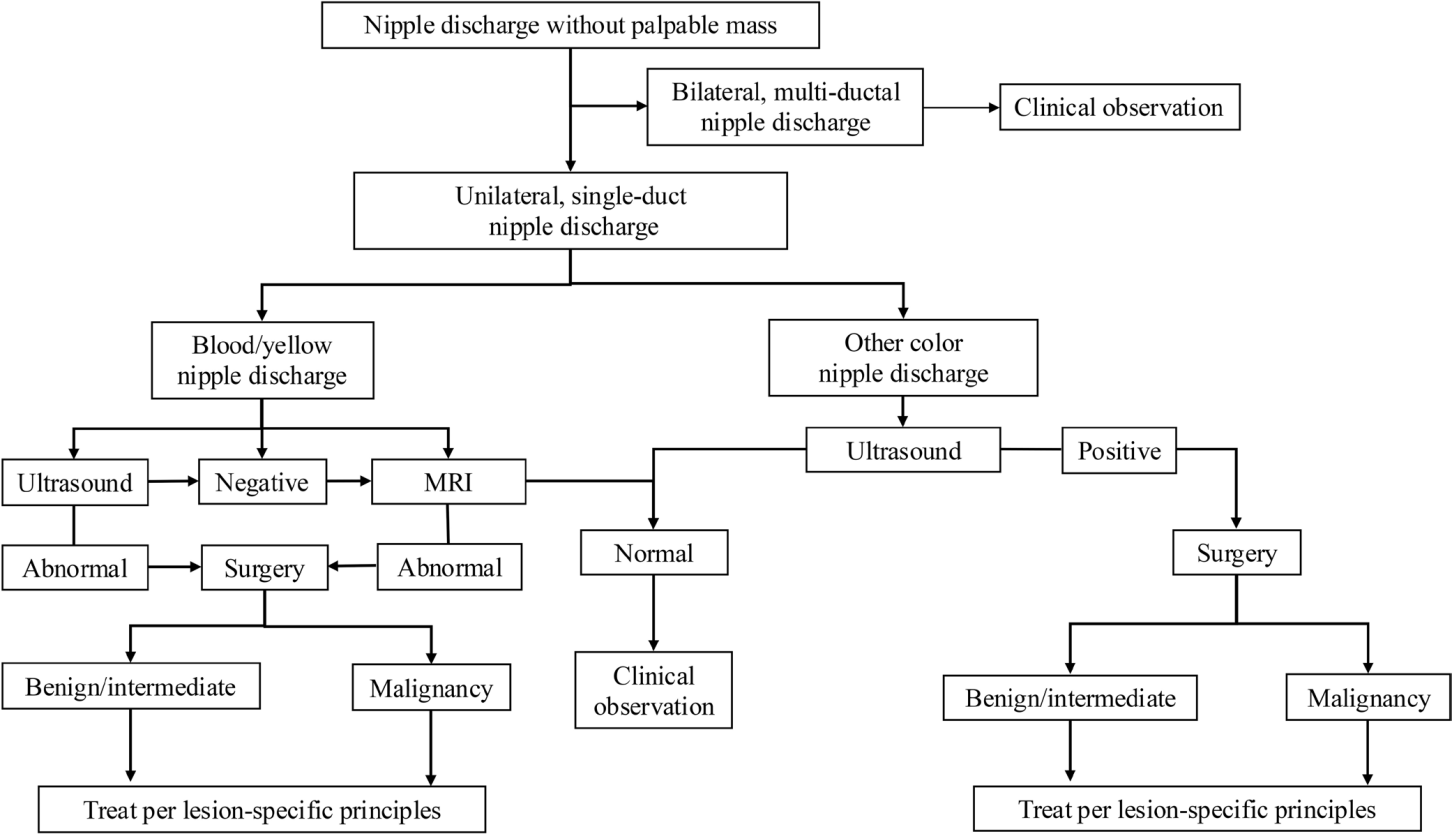
The diagnostic and surgical management for patients with nipple discharge without a clinically palpable mass.

According to the current study and previous guidelines, a diagnostic and surgical management for patients with nipple discharge without clinically palpable mass was thus formulated, as shown in (Figure 4). Bloody and yellow discharges were highly related to intraductal lesions, and ultrasound and MRI were beneficial in detecting suspicious lesions. Breast MRI aided in the selection of surgical incisions and the determination of resection scope. Mammography is greatly influenced by glandular density and has low diagnostic sensitivity for patients with nipple discharge without palpable masses. Mammograph positivity was not an independent risk factor for pathological positivity. In the study, it is recommended that patients with bloody or yellow discharge undergo ultrasound and MRI examinations, and judge whether the surgery is necessary according to the evaluation results. Patients with other colored discharges are suggested to undergo ultrasound. If the evaluation is positive, surgical intervention could be considered. If negative, a regular follow‐up could be conducted. Patients > 50 years of age were found to be at greater risk of breast cancer both in this study and in previous studies, so this population of patients should be followed more closely.

This study has several limitations. First, the retrospective, single‐center design introduces inherent selection bias; because our cohort exclusively comprised patients who underwent surgical intervention, the findings may not fully generalize to patients managed conservatively. Second, incomplete clinical or imaging data may have affected the accuracy of diagnostic performance assessment and multivariate risk‐factor analysis. Third, although MRI showed superior diagnostic sensitivity and high concordance with surgical localization, the results were not externally validated in an independent cohort. Therefore, the applicability of these findings to other populations, imaging protocols, and surgical practices remains uncertain. Prospective multicenter studies with standardized imaging protocols and external validation cohorts are warranted to confirm these findings and optimize risk stratification and surgical management.

## 5 Conclusion

In patients with nipple discharge without clinically palpable mass, MRI has a higher diagnostic sensitivity for intraductal lesions and breast cancer compared to ultrasound and mammography and is beneficial for assessing the location and the extent of lesions. Bloody or yellow discharge, positive ultrasound, and positive MRI are independent risk factors for intraductal lesions and may aid in selecting surgical indications in this population.

## Author Contributions

**Yang Yang:** conceptualization, data curation, formal analysis, methodology, project administration, resources, visualization, writing – original draft. **Siyuan Wang:** resources. **Miao Liu:** conceptualization, data curation, methodology, project administration, resources, supervision, validation, writing – review and editing. **Houpu Yang:** resources. **Lei Chen:** data curation, resources. **Shu Wang:** resources, supervision. **Dingbao Chen:** data curation, resources. **Jiaxuan Li:** investigation, validation, visualization. **Ting Lin:** formal analysis, investigation, visualization. **Fei Xie:** resources.

## Funding

The authors have nothing to report.

## Ethics Statement

The study protocol was approved by the Ethics Committee of Peking university people's Hospital.

## Conflicts of Interest

The authors declare no conflicts of interest.

## Supporting information


**Table S1:** Correlation of clinical characteristics and malignancy in patients with non‐palpable nipple discharge.

**Table S2:** Correlation of clinical characteristics and intraductal lesions in patients with non‐palpable nipple discharge.

**Table S3:** Correlation between positive imaging and pathological positivity.

**Table S4:** Correlation between positive imaging and pathological malignancy.

## Data Availability

The data that support the findings of this study are available from the corresponding author upon reasonable request.
