# Correction to “Clinical and Imaging Characteristics in the Diagnosis and Surgical Management of Nipple Discharge Without Clinically Palpable Masses: A Retrospective Cohort Study”

**DOI:** 10.1111/1759-7714.70395

**Published:** 2026-10-21

Y. Yang , T. Lin , J. Li , et al., “Clinical and Imaging Characteristics in the Diagnosis and Surgical Management of Nipple Discharge Without Clinically Palpable Masses: A Retrospective Cohort Study,” Thoracic Cancer 17, no. 12 (2026): e70332, 10.1111/1759-7714.70332.PMC1329155242338287

In the originally published version of this article, Lei Chen was inadvertently omitted as a co‐corresponding author.

The corresponding author information should have included the following:

Lei Chen (chenlei571@sina.com)

The article has been corrected online.

The authors apologize for this error.

