# Supplementary material for: Clinical and Imaging Characteristics in the Diagnosis and Surgical Management of Nipple Discharge Without Clinically Palpable Masses: A Retrospective Cohort Study
Source: Thorac Cancer. 2026 Jun 24;17(12):e70332. doi: 10.1111/1759-7714.70332 (PMC13291552; doi:10.1111/1759-7714.70332)
Supplement: Supplementary file 1 — Table S1: Correlation of clinical characteristics and malignancy in patients with non‐palpable nipple discharge. [file TCA-17-e70332-s001.docx]

Supplementary table S1. Analysis of correlation between clinical characteristics and maliganancy in patients with nipple discharge without palpable mass

| Characteristics | Malignancy   n (%) | Non-malignancy  n (%) | *P* value |
| --- | --- | --- | --- |
| No. of patients | 146 (100) | 530 (100) |  |
| Age (years) |  |  | ＜0.001 |
| ≤50 | 47(32.19) | 317（59.81） |  |
| ＞50 | 99(67.81) | 213（40.19） |  |
| Menstrual status |  |  | ＜0.001 |
| Pre-menopause | 51(34.93) | 326（61.51） |  |
| Post-menopause | 95(65.07) | 204（38.49） |  |
| Ductal involvement |  |  | 0.063 |
| Single duct | 143(97.95) | 499（94.15） |  |
| Multiple ducts | 3(2.05) | 31（5.85） |  |
| Color |  |  | ＜0.001 |
| Bloody | 89(60.96) | 233（43.96） |  |
| Yellow | 48(32.88) | 158（29.81） |  |
| Others† | 9(6.16) | 139（26.23） |  |
| Duration of the disease (month) |  |  | 0.069 |
| ≤1 | 49(33.56) | 154（29.06） |  |
| 1-3 | 38(26.03) | 99（18.68） |  |
| 3-12 | 41(28.08) | 189（35.66） |  |
| ＞12 | 18(12.33) | 88（16.60） |  |

† Others include serous, white nipple discharge. *P* value was determined by Chi-squared test
