# Supplementary material for: Clinical and Imaging Characteristics in the Diagnosis and Surgical Management of Nipple Discharge Without Clinically Palpable Masses: A Retrospective Cohort Study
Source: Thorac Cancer. 2026 Jun 24;17(12):e70332. doi: 10.1111/1759-7714.70332 (PMC13291552; doi:10.1111/1759-7714.70332)
Supplement: Supplementary file 2 — Table S2: Correlation of clinical characteristics and intraductal lesions in patients with non‐palpable nipple discharge. [file TCA-17-e70332-s004.docx]

Supplementary table S2. Analysis of correlation between clinical characteristics and intraductal lesions in patients with nipple discharge without palpable mass

| Characteristics | Pathologically positive group  N (%) | Pathologically negative group  n (%) | *P* value |
| --- | --- | --- | --- |
| No. of patients | 512 (100) | 164 (100) |  |
| Age (years) |  |  | 0.003 |
| ≤50 | 259（50.59） | 105（64.02） |  |
| ＞50 | 253（49.41） | 59（35.98） |  |
| Menstrual status |  |  | 0.001 |
| Pre-menopause | 267（52.15） | 110（67.07） |  |
| Post-menopause | 245（47.85） | 54（32.93） |  |
| Ductal involvement |  |  | ＜0.001 |
| Single duct | 497（97.07） | 145（88.41） |  |
| Multiple ducts | 15（2.93） | 19（11.59） |  |
| Color |  |  | ＜0.001 |
| Bloody | 294（57.42） | 28（17.07）^#^ |  |
| Yellow | 160（31.25） | 46（28.05）^*^ |  |
| Other† | 58（11.33） | 90（54.88）^*#^ |  |
| Duration of the disease (month) |  |  | 0.602 |
| ≤1 | 151（29.49） | 52（31.71） |  |
| 1-3 | 109（21.29） | 28（17.07） |  |
| 3-12 | 175（34.18） | 55（33.54） |  |
| ＞12 | 77（15.04） | 29（17.68） |  |

† Others include serous, white nipple discharge. *P* value by Chi-squared test
