# Supplementary material for: Clinical and Imaging Characteristics in the Diagnosis and Surgical Management of Nipple Discharge Without Clinically Palpable Masses: A Retrospective Cohort Study
Source: Thorac Cancer. 2026 Jun 24;17(12):e70332. doi: 10.1111/1759-7714.70332 (PMC13291552; doi:10.1111/1759-7714.70332)
Supplement: Supplementary file 3 — Table S3: Correlation between positive imaging and pathological positivity. [file TCA-17-e70332-s002.docx]

Supplementary table S3. Analysis of the correlation between positive imaging and pathologically positivity

| Imaging | Pathologically positivity | Pathologically negativity | Total | *P* value |
| --- | --- | --- | --- | --- |
| Ultrasound BI-RADS ≥4 | 309 | 45 | 354 | ＜0.001 |
| Ultrasound BI-RADS ≤3 | 170 | 110 | 280 |  |
| Total | 479 | 155 | 634 |  |
| Mammography BI-RADS ≥4 | 118 | 28 | 146 |  |
| Mammography BI-RADS ≤3 | 328 | 111 | 439 | 0.133 |
| Total | 446 | 139 | 585 |  |
| MRI BI-RADS ≥4 | 195 | 40 | 235 |  |
| MRI BI-RADS ≤3 | 45 | 42 | 87 | ＜0.001 |
| Total | 240 | 82 | 322 |  |
| Ultrasound/MRI BI-RADS ≥4  (Parallel combination model) | 210 | 49 | 259 | ＜0.001 |
| Ultrasound/MRI BI-RADS ≤3  (Parallel combination model) | 18 | 30 | 48 |  |
| Total | 228 | 79 | 307 |  |
| Ultrasound/MRI BI-RADS ≥4  (Serial combination model) | 135 | 8 | 143 | ＜0.001 |
| Ultrasound/MRI BI-RADS ≤3  (Serial combination model) | 93 | 71 | 164 |  |
| Total | 228 | 79 | 307 |  |

BI-RADS: Breast Imaging Reporting and Data System. MRI: magnetic resonance imaging. Parallel combination: a combined positive result was defined as a BI-RADS ≥4 finding on either ultrasound or MRI. Serial combination: a combined positive result was defined as a BI-RADS ≥4 finding on both ultrasound and MRI. *P* value was determined by Chi-squared test.
