# Supplementary material for: Clinical and Imaging Characteristics in the Diagnosis and Surgical Management of Nipple Discharge Without Clinically Palpable Masses: A Retrospective Cohort Study
Source: Thorac Cancer. 2026 Jun 24;17(12):e70332. doi: 10.1111/1759-7714.70332 (PMC13291552; doi:10.1111/1759-7714.70332)
Supplement: Supplementary file 4 — Table S4: Correlation between positive imaging and pathological malignancy. [file TCA-17-e70332-s003.docx]

Supplementary table S4. Analysis of the correlation between positive imaging and pathological malignancy

| Imaging | Malignancy | Non-malignancy | Total | P value |
| --- | --- | --- | --- | --- |
| Ultrasound BI-RADS ≥4 | 101 | 173 | 274 | ＜0.001 |
| Ultrasound BI-RADS ≤3 | 35 | 325 | 360 |  |
| Total | 136 | 498 | 634 |  |
| Mammography BI-RADS ≥4 | 65 | 82 | 147 |  |
| Mammography BI-RADS ≤3 | 64 | 374 | 438 | ＜0.001 |
| Total | 129 | 456 | 585 |  |
| MRI BI-RADS ≥4 | 62 | 140 | 202 |  |
| MRI BI-RADS ≤3 | 9 | 111 | 120 | ＜0.001 |
| Total | 71 | 251 | 322 |  |
| Ultrasound/MRI BI-RADS ≥4  (Parallel combination model) | 69 | 190 | 259 | ＜0.001* |
| Ultrasound/MRI BI-RADS ≤3  (Parallel combination model) | 0 | 48 | 48 |  |
| Total | 69 | 238 | 307 |  |
| Ultrasound/MRI BI-RADS ≥4  (Serial combination model) | 55 | 88 | 143 | ＜0.001 |
| Ultrasound/MRI BI-RADS ≤3  (Serial combination model) | 14 | 150 | 164 |  |
| Total | 69 | 238 | 307 |  |

BI-RADS: Breast Imaging Reporting and Data System. MRI: magnetic resonance imaging. Parallel combination: a combined positive result was defined as a BI-RADS ≥4 finding on either ultrasound or MRI. Serial combination: a combined positive result was defined as a BI-RADS ≥4 finding on both ultrasound and MRI. *P* value was determined by Chi-squared test. * Fisher's exact test was applied due to a zero cell count.
